# A Facile One-Pot Preparation and Properties of Nanocellulose-Reinforced Ionic Conductive Hydrogels

**DOI:** 10.3390/molecules28031301

**Published:** 2023-01-30

**Authors:** Xinmin Huang, Yaning Wang, Yifei Wang, Lianhe Yang

**Affiliations:** 1 College of Textile & Clothing, Yancheng Institute of Technology, Yancheng 224051, China; 2 School of Textile & Science Engineering, Tiangong University, Tianjin 300387, China

**Keywords:** cellulose nanofiber, polyvinyl alcohol, ionic conductive hydrogels, sensors

## Abstract

Nanocellulose-reinforced ionic conductive hydrogels were prepared using cellulose nanofiber (CNF) and polyvinyl alcohol (PVA) as raw materials, and the hydrogels were prepared in a dimethyl sulfoxide (DMSO)/water binary solvent by a one-pot method. The prepared hydrogels were characterized by scanning electron microscopy (SEM) and Fourier transform infrared spectroscopy (FTIR). The mechanical properties, electrical conductivity, and sensing properties of the hydrogels were studied by means of a universal material testing machine and LCR digital bridge. The results show that the ionic conductive hydrogel exhibits high stretchability (elongation at break, 206%) and firmness (up to 335 KPa). The tensile fracture test shows that the hydrogel has good properties in terms of tensile strength, toughness, and elasticity. The hydrogel as a conductor medium is assembled into a self-powered strain sensor and the open-circuit voltage can reach 0.830 V. It shows good sensitivity in the bend sensing testing, indicating that the hydrogel has good sensing performance. The water retention and anti-freezing performance experiments show that the addition of dimethyl sulfoxide solvents can effectively improve the anti-freezing and water retention properties of hydrogels.

## 1. Introduction

In recent years, wearable smart sensors have attracted growing attention in advanced fields, such as electronic skin and health monitoring [1]. At present, traditional sensors based on inorganic or metal materials in the market usually have excellent electrical properties, durability, and compatibility. However, due to their stiffness, brittleness, hydrophobicity, and weak tensile properties, traditional sensors are not suitable for wearable sensors used for human motion detection [2,3]. Three-dimensional conductive hydrogels with excellent flexibility have received considerable attention as emerging materials with substantial applications as electronic skins, flexible wearable devices, multifunctional sensors, and so on.

Polyvinyl alcohol (PVA) is a cheap biopolymer, which is widely used in many fields such as bioengineering, modern medicine, textiles, and garments [4]. It is easily soluble in biological media and can use various cross-linking agents to form stable new hydrogels based on polyvinyl alcohol [5,6]. In general, the addition of nanomaterials to hydrogel systems is an effective and practical way to improve the mechanical strength of hydrogels. Cellulose nanofiber derived from the most abundant native renewable biomass has unique and promising properties, such as high crystallinity, aspect ratio, specific surface area, and tensile strength [7]. This material is non-toxic, harmless, lightweight, and has good biocompatibility. More importantly, the surface of nanofibers is rich in hydroxyl groups, with high elastic modulus, stiffness, and low thermal expansion coefficient, which can be stably dispersed in water-soluble polymers or as matrix composites dispersed in water [8,9,10,11,12,13]. In this regard, cellulose is often combined with conductive fillers to make conductive nanocomposite complexes, which play an important role in toughening, crosslinking, and acting as network support with excellent mechanical properties. Carbon nanotubes (CNTs) have been widely used to prepare conductive hydrogels due to their excellent mechanical properties and electrical conductivity. However, CNTs are not easily dispersed in water under the action of van der Waals force. When CNF interacts with a CNT, due to the strong nonpolar interaction of cellulose with its surface energy, a significant van der Waals interaction occurs. Moreover, when CNTs are adsorbed onto CNFs, all the water bound to CNFs will be released, resulting in a large increase in entropy. Considering the large specific surface area of CNFs, this may imply that the driving force for CNFs to disperse CNTs should be the overall gain in entropy and the highly nonpolar interaction between the two nanoparticles [14,15]. Ge et al. [16] designed a self-healing composite hydrogel with antioxidant and antibacterial activity using nanocellulose and tannic acid (TA) as functional additives. Due to the combined dynamic borate ester bonding between polyvinyl alcohol–borax and multi-hydrogen bonding between different components, excellent mechanical stability, stretchability, and rapid self-healing ability were realized in one system. Bai et al. [17] proposed a strategy using ethanol to dynamically adjust the hydrogen bond crosslinking between polyvinyl alcohol and tannic acid to prepare hydrogel coatings. Because ethanol dynamically regulates the hydrogen bonds between PVA and TA in water, a uniform hydrogel coating is formed on the surface of the porous substrate. The obtained hydrogel coating exhibits ultra-high strength, swelling volume stability, and excellent oil–water separation efficiency. Yang et al. [18] used cellulose nanofibers (CNF) as a dispersant to promote the uniform dispersion of multi-walled carbon nanotubes (MWCNTs) in hydrogels. Hamedi et al. [15] used well-dispersed CNF/CNT nanohybrids to fabricate conductive hydrogels for wearable flexible sensors and intelligent electronic skins. Li et al. [19] grafted lignin-based carbon (LC) as the conductive filler and cellulose nanofibrils (CNF), carboxymethyl chitosan (CMC), and polyvinyl alcohol (PVA) as the polymer matrix to prepare a conductive hydrogel, which endowed it with a tensile strength of 133 kPa. Wei et al. [20] synthesized by a one-step acrylamide polymerization in the presence of cellulose nanofiber (CNF), templated carbon nanotube (CNT) hybrids, and glycerol–water binary solvent, which synergistically endows the organohydrogel with excellent tensile strength (≈119.2 kPa).

Due to the large amount of water contained in a hydrogel, it is easy to freeze and evaporate, which dramatically restricts the practical application of hydrogels. Most conventional hydrogels have poor environmental stability because of the low-temperature-induced freezing problem. The addition of organic solvents is a strategy to improve the anti-freezing performance of hydrogels. Dimethyl sulfoxide (DMSO) is widely used to cryopreserve cells and has recently been used to prepare freeze-resistant hydrogels [21]. 

Therefore, multifunctional hydrogels with good mechanical strength, anti-freezing properties, moisture retention, and long-term stability are highly desirable for human motion monitoring. In this experiment, a high-strength, anti-freezing, and stretchable nanocellulose-enhanced ionic conductive hydrogel was prepared by a one-pot method using CNF and polyvinyl alcohol (PVA) as raw materials, adding dimethyl sulfoxide (DMSO), AlCl_3_, and carbon nanotubes. Dimethyl sulfoxide (DMSO) was added to improve the anti-freezing performance and water retention performance of the hydrogel, and AlCl_3_ and carbon nanotubes were incorporated to improve the conductivity of the hydrogel. The microstructure of the prepared hydrogel was characterized by scanning electron microscopy (SEM) and Fourier transform infrared spectroscopy (FTIR). The mechanical properties, electrical conductivity, anti-freezing properties, water retention properties, and sensing properties of the hydrogels were studied by means of testing methods such as universal material testing machines and the LCR digital bridge. The results show that the prepared hydrogel has good electrical conductivity, anti-freezing performance, and good mechanical properties. At the same time, the hydrogel can be applied to human motion detection and has the potential to be used in flexible sensors.

## 2. Results and Discussion

### 2.1. Surface Topography

**Figure 1:**
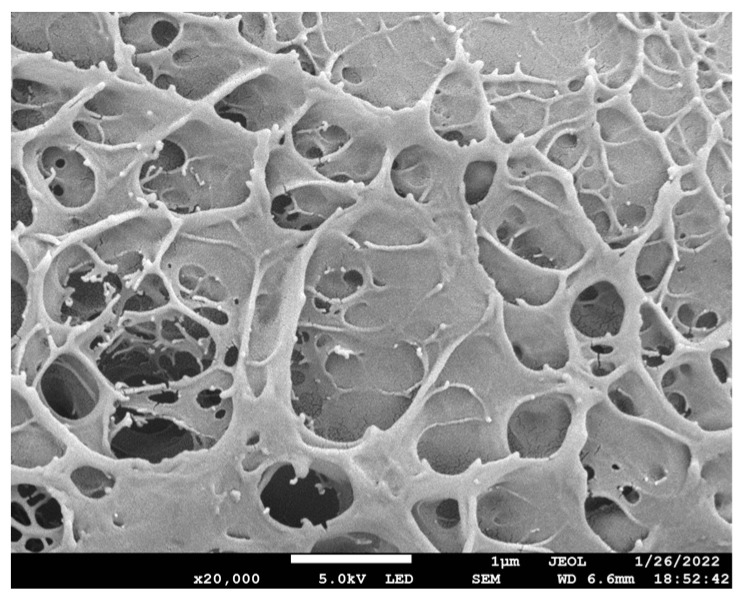
SEM image of the PVA-CNF hydrogel.

As shown in Figure 1, the three-dimensional porous network structure of the hydrogel can be seen. The pore size is between a few hundred nanometers and a micron, making it easier for some ions to enter the internal network of the hydrogel. This kind of ordered porous structure lays a foundation for the establishment of conductive pathways. Due to the three-dimensional porous network structure of the hydrogel, it provided a pathway for ion transmission and improved the conductivity of the hydrogel [22].

**Figure 2:**
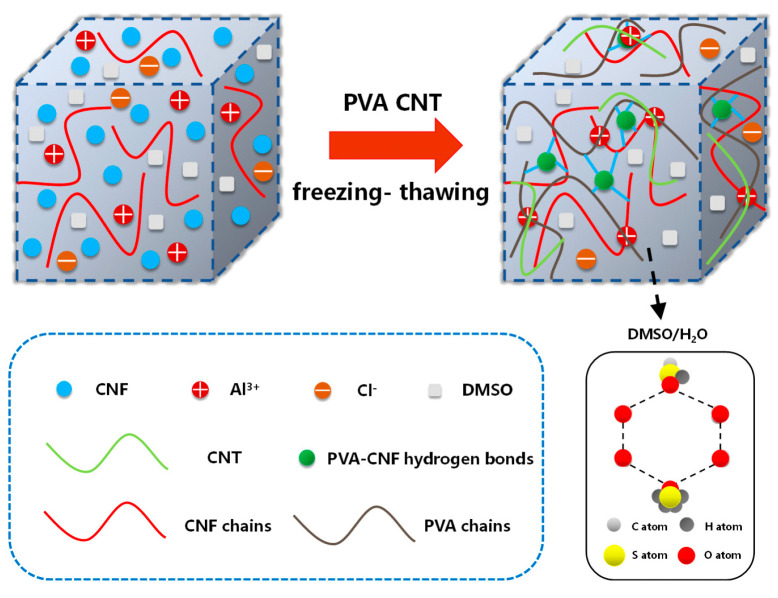
Schematic diagram of the preparation process of the PVA/CNF hydrogel.

The PVA-CNF hydrogels were fabricated via a one-pot method. As shown in the schematic diagram (Figure 2), the CNF was used as a reinforcement agent and a strong hydrogen bonding crosslinker in the system. Al^3+^ ions were introduced into the hydrogel to act as conductive ions.

### 2.2. Infrared Spectroscopy Analysis

**Figure 3:**
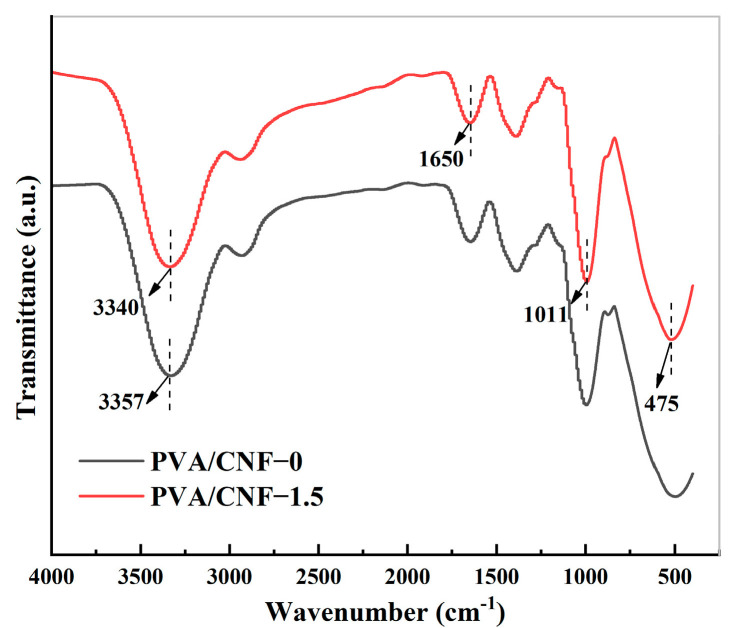
FT-IR spectra of PVA/CNF-0 and PVA/CNF-1.5 dehydrated hydrogels.

As shown in Figure 3, the hydrogel exhibited a broad and strong peak at 3357 cm^−1^, which was attributed to the intermolecular and intramolecular hydrogen bonds between PVA and CNF. It can be seen from the spectra that the peak shifts to lower wave numbers as the CNF content increases from 0 to 1.5%. The PVA-CNF hydrogel exhibits absorption peaks at 1650 and 475 cm^−1^ associated with the enhanced C–H bending vibrations of the DMSO, while the stretching absorption peak at 1011 cm^−1^ is attributed to the S = O characteristic peak of DMSO. The DMSO forms strong hydrogen bond cross-links with water molecules and also enhanced the mechanical properties of the hydrogels. The CNF may also be induced to produce coordination bonds under the reaction of both parties, resulting in the formation of microcrystals in PVA to enhance the mechanical properties of the hydrogel. 

### 2.3. Mechanical Property Analysis

**Figure 4:**
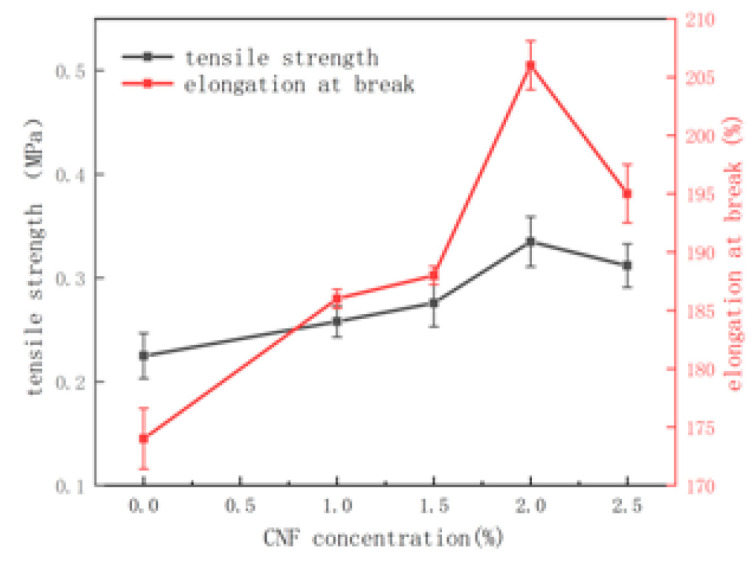
Tensile strength values and elongation at the break of hydrogels with different CNF contents.

The tensile stress–strain curves of hydrogels at room temperature were tested by a universal material testing machine (Figure 4), and the tensile stress of the hydrogel changes continuously with the increase in CNF content. When the content of CNF is added to 2%, the tensile strength and elongation at the break of the hydrogel are 0.335 MPa and 206%, respectively. When the content of CNF continues to increase, the tensile strength and elongation at the break of the hydrogel decrease significantly. As a polymer nano-reinforcement agent, CNF forms hydrogen bonds with PVA chains, which effectively improves the mechanical properties of the hydrogels [21]. However, a high CNF concentration has a negative impact on the mechanical properties of hydrogels, as it causes CNFs to aggregate or form more crystallites in the hydrogel of the polymer material, which leads to inhomogeneous distribution in the three-dimensional network structure and leads to the degradation of the mechanical properties.

**Figure 5:**
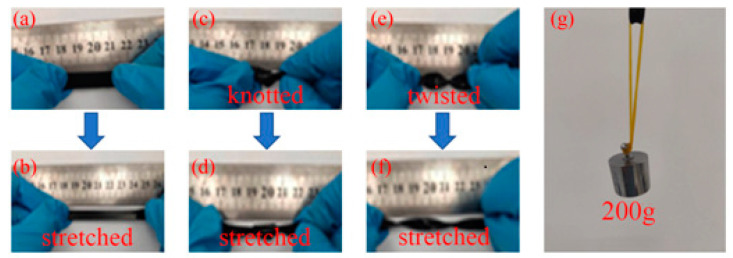
Mechanical properties of the hydrogel.: (**a**) initial hydrogel (**b**) stretched (**c**) knotted (**d**) stretched after knotted (**e**) twisted (**f**) stretched after twisted (**g**) hydrogel 200 g load demo.

As shown in Figure 5, the PVA-CNF hydrogel exhibits excellent flexibility and mechanical strength. The PVA-CNF hydrogel can withstand 200 g of weight without breaking (equivalent to 333 times its weight). The PVA-CNF hydrogel can be stretched to twice its distance without breaking, and can still be stretched twice its length without breaking in the case of knotting and twisting, which reflects the excellent mechanical properties of the PVA-CNF hydrogel.

### 2.4. Ionic Conductivity

**Figure 6:**
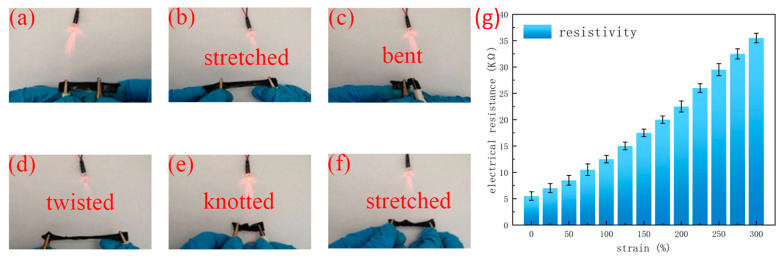
(**a**–**f**) Brightness change of LED lamp after hydrogel deformation. (**g**) Resistance change with the increase in tensile strain.

The ionic conductive hydrogel acts as a conductor in the circuit and is connected to a 3 V power supply (Figure 6a). The LED light can be lightened when the hydrogel is stretched, bent, twisted, and knotted (Figure 6b–f). The brightness of the LED light decreased as the strain increased, indicating that the resistance of the hydrogel increased with the stretching. As shown in Figure 6g, the digital bridge was used to analyze the resistance and the relative resistance change for the conductive hydrogel under various tensile strains. The results show that the resistance of the hydrogel increased gradually with the increase in strain. The main reason is that with the increase in tensile strain, the distance between the conductive segments inside the hydrogel network becomes longer due to the interference of external forces [23]. At the same time, the transmission of ions in the hydrogel pore structure is blocked, which leads to an increase in the resistance of the hydrogel.

**Figure 7:**
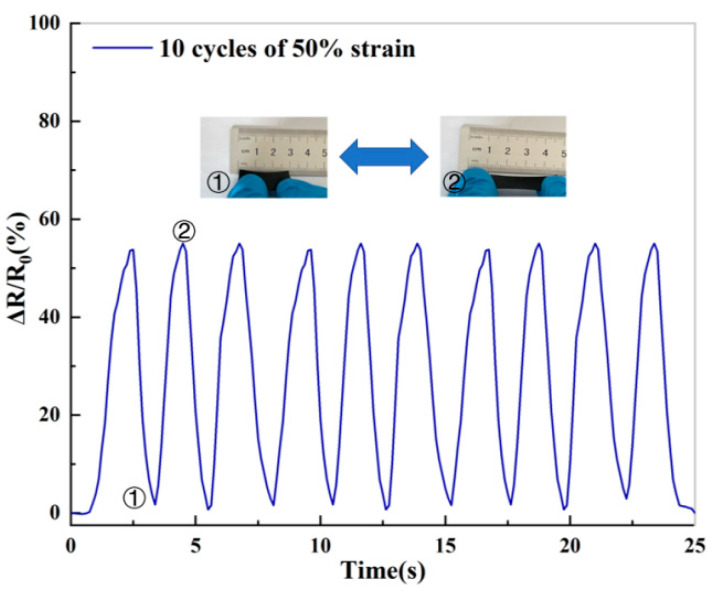
Relative resistance curves of the hydrogel at 50% strain. “①” denotes original length and “②” denotes 1.5 times the original length.

The ionic conductive hydrogel was stretched to 1.5 times its length and then restored to its original length. The cycle test was performed 10 times, and the change rate of the relative resistance of the hydrogel was recorded by an LCR digital bridge. The results are shown in Figure 7. In this process, the relative resistance changes repeatedly in a stable range and the relative resistance has no significant loss. When the hydrogel recovers the original length, the relative resistance also recovers the initial value, which indicates its potential application in strain sensors.

**Figure 8:**
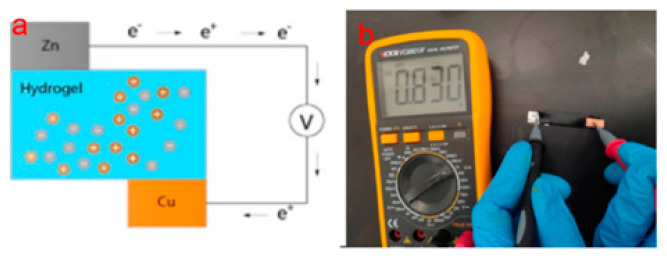
(**a**) Self-powered device schematic diagram. (**b**) Hydrogel as an electrolyte to form a self-powered device with a primary battery structure.

A self-powered strain sensor device based on a hydrogel battery was fabricated using a digital multimeter. The schematic diagram is shown in Figure 8a. The hydrogel, zinc foil, and copper foil were assembled into a primary battery structure using zinc foil as the negative electrode and copper foil as the positive electrode. Electrons flow continuously from the negative electrode to the positive electrode through a wire to form a potential difference. The self-powered device converts chemical energy into electrical energy. The test found that the generated voltage was about 0.830 V (Figure 8b). This self-powered sensor frees itself from the limitations of external power sources and expands the hydrogel’s potential application in wearable sensors.

**Figure 9:**
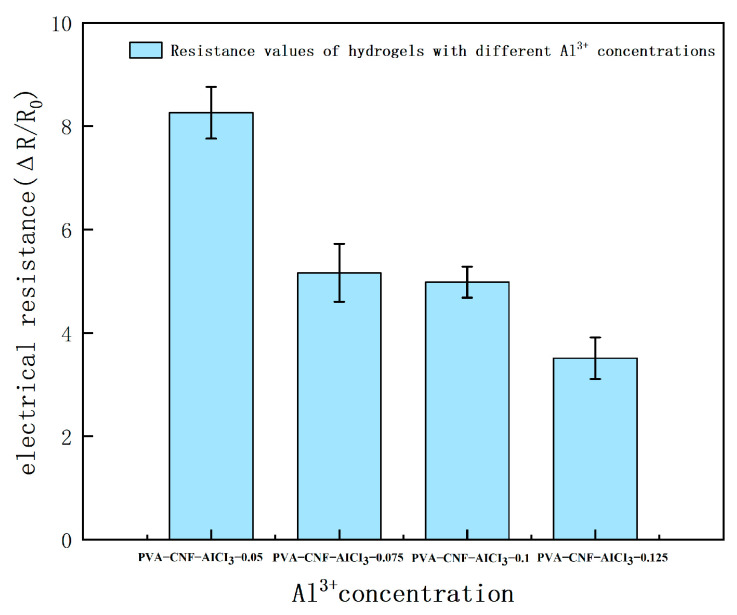
Relative resistance change of hydrogels with different Al^3+^ concentrations.

As shown in Figure 9, the resistance value of PVA-CNF hydrogels with different Al^3+^ ion concentrations changes under the test of a digital bridge. From the diagram, it can be seen that with the continuous increase in Al^3+^ concentration, the resistance value shows a downward trend, indicating that with the continuous increase in Al^3+^ concentration, the conductivity of the hydrogel is continuously improved. This is due to the continuous increase in Al^3+^ concentration, which will increase the efficiency of ion passage in the hydrogel, resulting in a decrease in the resistance of the hydrogel.

### 2.5. Sensing Performance

**Figure 10:**
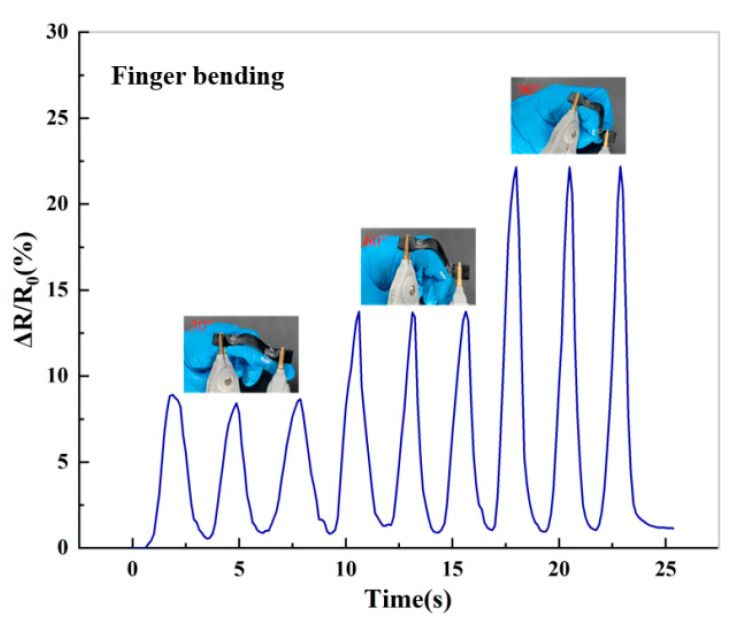
Relative resistance of hydrogel strain sensors at varying finger angles (30°, 60°, and 90°).

Figure 10 shows that the hydrogel could detect small finger movements with different angles. The resistance of the hydrogel changed with different bending angles. The relative resistance signal correspondingly increased with increasing angles of bending from 30° to 90° and instantly returned to the initial resistance when the finger returned to the initial degree. Interestingly, the resistance value of the hydrogel remained constant while holding the same bending angle of the forefinger. This indicates that this hydrogel-based sensor has high sensitivity. As the hydrogel elongates, the ion channels and electron channels are elongated, increasing the relative resistance change of the hydrogel. The results show that this hydrogel-based strain sensor has a certain potential application in wearable electronic devices.

**Figure 11:**
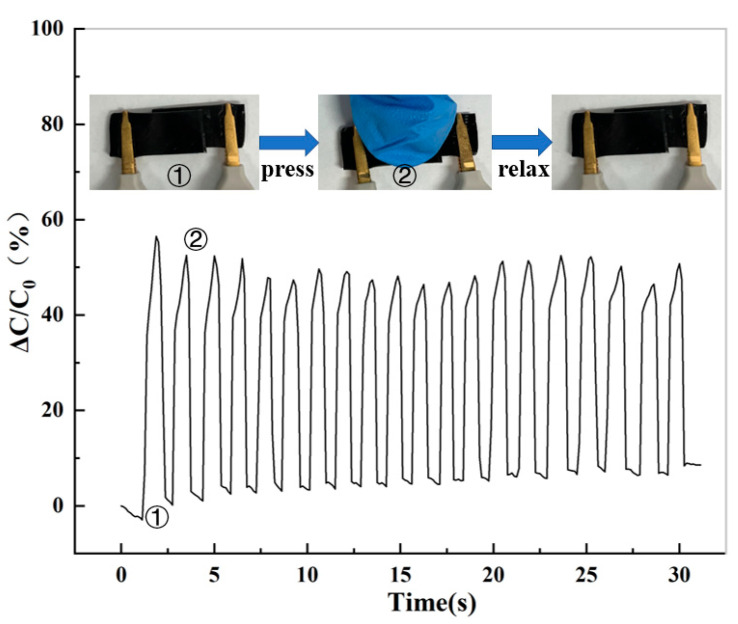
Variation of different pressure and relative resistance. “①” denotes release and “②” denotes press.

The cut hydrogel sample is connected by VHB tape to assemble a capacitor and connected to the instrument. The relative capacitance of the capacitor composed of the hydrogel is tested during the process of applying pressure to releasing pressure. As shown in Figure 11, it can be found that the relative capacitance shows a stable change in a range, indicating that the hydrogel has good pressure sensitivity, and at the same time, the change in pressure will also produce a corresponding relative capacitance change. The sensitivity of pressure reflected by the hydrogel can sensitively reflect the change in the pressure when worn on the human body, which provides feasibility for the application of the hydrogel to human intelligent wearables.

### 2.6. Anti-Freezing and Moisture Performance

**Figure 12:**
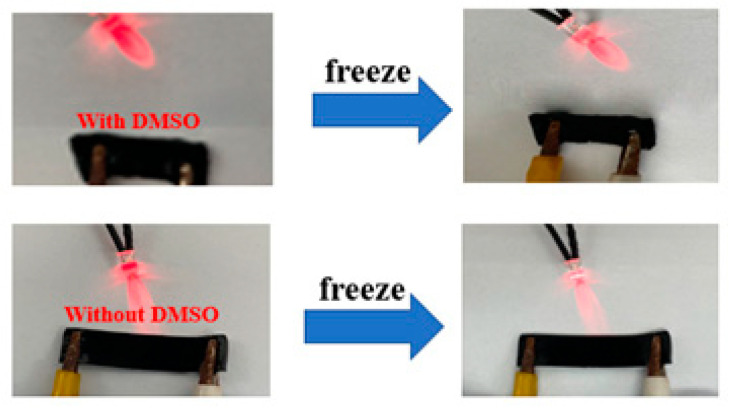
The brightness change of the LED lamp after 24 h of freezing.

Improving the low-temperature tolerance of hydrogel sensors is necessary for the use of flexible sensors using hydrogels as substrates at low temperatures [24]. The electrical conductivity of the hydrogels at low temperatures was also investigated. The more conductive the hydrogel is the less resistive it is. As shown in Figure 12, the PVA-CNF hydrogels were used to light an LED light in a circuit using a 3 V power source, which indicated its conductivity. The LED light brightness was decreased with increasing resistance. By analyzing the brightness of the LED lamp of the control hydrogel, it can be seen that the LED light intensity of these hydrogels was dim after freezing and the LED light brightness of the hydrogel without DMSO became dimmer. The above experimental results indicate that the conductivity of the hydrogel without DMSO is greatly affected. However, the conductivity of the hydrogel with DMSO was not much affected. Hydrogel with DMSO showed less deformation after freezing, and its toughness was better than the hydrogel without DMSO, indicating that the addition of DMSO can improve the freezing resistance of hydrogels. The reason why the hydrogel with DMSO shows a certain freezing resistance is mainly due to the strong hydrogen bonding force between the sulfinyl group in DMSO and water molecules, which makes the formation of ice crystals more difficult, and then makes the double network cross-linking between PVA and water molecules more solid, making the PVA-CNF composite hydrogel not easy to freeze and lose electrical properties [21].

**Figure 13:**
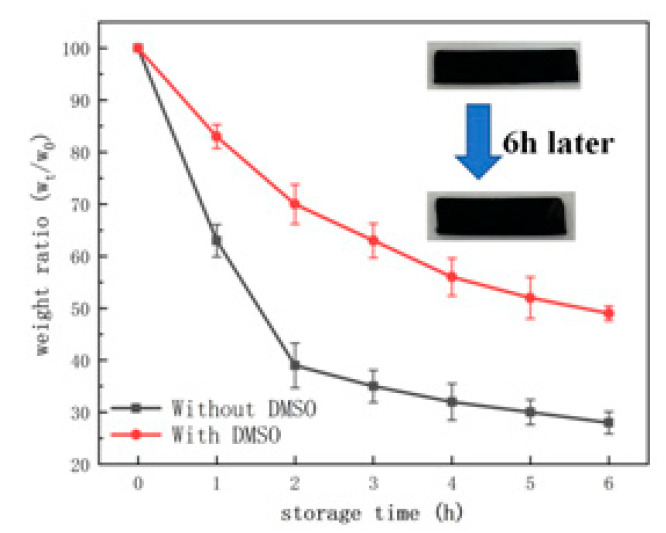
Mass change pattern and change curve of hydrogel before and after drying.

The water content inside the hydrogel will be lost during long-term use, which seriously affects the structure and properties of the hydrogel. Therefore, moisture performance is also an important factor in hydrogel strain sensors. Figure 13 shows the changes in the quality and morphology of the two hydrogels after drying in the oven for 6 h. It can be seen that the weight loss of the two gels is large because there is a large amount of free water on the surface of the initial hydrogel, which is easy to evaporate and causes mass loss. After 6 h, the weight loss of the hydrogel without DMSO was 70%, while that of the hydrogel with DSMO remained stable after 45% weight loss. This is due to the strong intermolecular force between the substances in the hydrogel and the water molecules, which inhibits the evaporation of water to a certain extent so that the hydrogel has better water retention performance. The addition of DMSO can inhibit the formation of ice crystals, further strengthen the hydrogen bonding force inside the hydrogel system, and slow down the speed of water loss to a certain extent [21].

### 2.7. Touch Properties of Hydrogels

**Figure 14:**
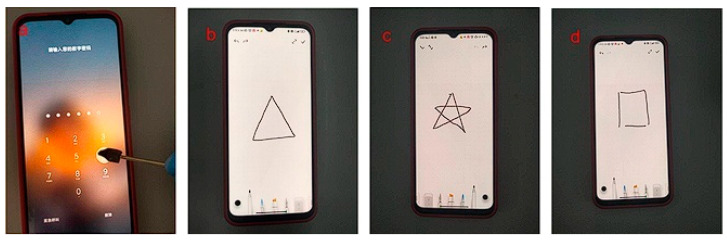
Electronic pen made from PVA-CNF hydrogel can unlock the desktop (**a**) and draw (**b**–**d**).

The PVA/CNF hydrogel and non-conductive thin rod can be assembled into an electronic pen, which can unlock the desktop (Figure 14a) and draw graphics on the screen (Figure 14b–d). The ionic conductive hydrogel can be used as an electronic pen because the free ions between the ionic conductive hydrogel and the touch screen can form a coupling capacitance, reflecting the potential application of the hydrogel in the field of human–computer interaction.

## 3. Experimental Section

### 3.1. Materials

Polyvinyl alcohol-1750 (PVA) was purchased from Sinopharm Chemical Reagent Co., Ltd., Huangpu, Shanghai, China. Pulp Cellulose Nanofiber (CNF) was purchased from Guilin Qi Hong Technology Co., Ltd., Guilin, Guangxi, China. Aluminum chloride hexahydrate (AlCl_3_·6H_2_O), dimethyl sulfoxide (DMSO), and carbon nanotubes were supplied by Shanghai Titan Scientific Co., Ltd., Xuhui, Shanghai, China. All the reagents were used directly without further purification.

### 3.2. Preparation of the PVA-CNF Hydrogels

The conductive hydrogels were constructed as follows. Firstly, 20 g of a certain concentration of cellulose nanofiber (CNF) suspension was prepared, then 20 g of DMSO solvent was added to the CNF suspension, and the mixed solution was stirred on a magnetic stirrer for 30 min to make it fully mixed. An amount of 3.6 g of polyvinyl alcohol (PVA) particles and 1.2 g of AlCl_3_·6H_2_O and carbon nanotube (0.18 g) particles were added to the above mixture and continued to be stirred uniformly with a magnetic stirrer. Then, the mixture was continuously and vigorously stirred at 120 °C until the PVA was completely dissolved. Finally, the PVA/CNF mixture was settled in an oil bath without stirring to remove bubbles, and then transferred to a PTFE mold and followed by freezing in the refrigerator at 20 °C for 12 h. The freezing–thawing process was repeated for three cycles to further enhance the mechanical properties of the hydrogel. The hydrogels with CNF concentrations of 0 wt%, 1 wt%, 1.5 wt%,2 wt%, 2.5 wt% were denoted as PVA-CNF-0, PVA-CNF-1, PVA-CNF-1.5, PVA-CNF-2, and PVA-CNF-2.5 hydrogels. The thickness of the hydrogel was controlled by weighing the same mass of solution.

The PVA-CNF hydrogels with different Al^3+^ concentrations were prepared by changing AlCl_3_ concentration to 0.05 mol (0.24 g/20 mL), 0.075 mol (0.36 g/20 mL), 0.1 mol (0.48 g/20 mL), and 0.125 mol (0.6 g/20 mL). These hydrogels were denoted as PVA-CNF-AlCl_3_-0.05, PVA-CNF-AlCl_3_-0.075, PVA-CNF-AlCl_3_-0.1, and PVA-CNF-AlCl_3_-0.125 hydrogels. The compositions of the PVA-CNF hydrogels are listed in Table 1. 

### 3.3. Scanning Electron Microscope

The PVA-CNF hydrogel sample was processed by freeze-drying to obtain a dehydrated hydrogel. The surface morphology of the PVA-CNF ionic conductive hydrogels was observed by using a JEM-2200FS scanning electron microscope. The hydrogel samples were dried before testing and sprayed with gold to study the pore structure of the hydrogels.

### 3.4. Fourier Transform Infrared Spectroscopy

The nanocellulose-reinforced ionic conductive hydrogel samples were dehydrated by freeze-drying, and then the chemical structure of the dehydrated hydrogel samples was tested by CX-9600 Fourier Infrared Spectroscopy (FTIR, Wuxi Chuangxiang Analytical Instrument Co., Ltd., Jiangsu China). The samples were analyzed by spectral analysis according to the standard. The wave number range was 300–800 nm, the scanning speed was 500 nm/min, and the interval was 20 nm.

### 3.5. Mechanical Properties

The above-prepared nanocellulose reinforced ionic conductive hydrogel samples of different specifications were cut respectively to make 400 mm × 80 mm × 2 mm sample strips, and then the mechanical properties of the sample strips were tested by a universal material testing machine. The loading speed of the testing machine was 20 mm/min.

### 3.6. Ionic Conductive Property

The voltage of the hydrogel self-powered strain sensor was recorded by a digital multimeter, and the impedance value data of the hydrogel were obtained by a digital bridge tester (LCR TH2822A).

The ionic conductive hydrogel sample (40 × 10 × 2 mm) was quantitatively stretched and tested by a universal material testing machine. The relative resistance change of the hydrogel under tensile strain was tested by an LCR digital bridge tester (LCR TH2822A). The relative change in resistance (ΔR/R_0_, where ΔR = R − R_0_) was calculated.

### 3.7. Anti-Freezing Test

The 1 wt% DMSO-free PVA-CNF hydrogel was made and cut into a sample of 40 mm × 100 mm × 5 mm. At the same time, the prepared hydrogel sample was cut out to make a sample of 40 mm × 100 mm × 5 mm. It was frozen for 24 h in a refrigerator below *−*20 °C and then taken out for testing.

### 3.8. Moisture Retention Property Measurements

All samples were cut into a shape of 20 mm × 4 mm × 1 mm, and the hydrogel samples were placed in an oven at 50 °C for 6 h. The weight of the hydrogel W_t_ was measured every 1 h, and the initial mass was W_0_.

The calculation formula for the water retention rate is as follows:

$$Water\;retention\;rate=W_{t}/W_{0}\times 100\%$$

### 3.9. Sensor Characterization and Test

The above-prepared nanocellulose-reinforced ionic conductive hydrogel samples were cut to make a 40 mm × 100 mm × 5 mm sample strip. The sample strip was tied to the index finger with tape and the angle of the finger was bent (0° to 90°). The resistance of the hydrogel under different strains was recorded by an LCR digital bridge tester. The relative resistance change formula is (R − R_0_)/R_0_ × 100%, where R is the dynamic resistance under different strains, and R0 is the initial state resistance without strain.

## 4. Conclusions

In summary, ionic conductive hydrogels were prepared by a one-pot method using CNF, PVA, CNT, DMSO, and AlCl_3_. The surface of PVA/CNF hydrogels has a three-dimensional porous network structure, and the pore size is between several hundred nanometers and one micron. At the same time, the gel has good tensile properties and can withstand 333 times its weight without breaking. With the increase in CNF content, the tensile strength and elongation at the break of the hydrogel increased. When the content of CNF was added to 2%, the tensile strength and elongation at the break of the hydrogel were 0.335 MPa and 206%, respectively. With the continuous increase in CNF content, the tensile strength and elongation at break began to decrease. The hydrogel has a certain conductivity under stretching, twisting, and bending, and the CNF hydrogel can be assembled as an electrolyte into a self-powered battery with a battery voltage of 0.830 V. The simple sensor made of nanocellulose-reinforced ionic conductive hydrogel has a stable response to strain stress. The hydrogel can also be easily assembled into an electronic pen, and the assembled electronic pen has good touch performance. Based on these results, we believe that the developed hydrogel has good potential for high-performance wearable devices in low-temperature environments.

## Figures and Tables

**Table 1:** Compositions of the hydrogels.

Sample	PVA	CNF	AlCl_3_∙6 H_2_O	DMSO	CNT
PVA-CNF-0	3.6 g	20 g (0.0 wt% CNF)	1.2 g	20 g	0.18 g
PVA-CNF-1	3.6 g	20 g (0.1 wt% CNF)	1.2 g	20 g	0.18 g
PVA-CNF-1.5	3.6 g	20 g (0.15 wt% CNF)	1.2 g	20 g	0.18 g
PVA-CNF-2	3.6 g	20 g (0.2 wt% CNF)	1.2 g	20 g	0.18 g
PVA-CNF-2.5	3.6 g	20 g (0.25 wt% CNF)	1.2 g	20 g	0.18 g
PVA-CNF-AlCl3-0.05	3.6 g	20 g (0.2 wt% CNF)	0.24 g	20 g	0.18 g
PVA-CNF-AlCl3-0.075	3.6 g	20 g (0.2 wt% CNF)	0.36 g	20 g	0.18 g
PVA-CNF-AlCl3-0.1	3.6 g	20 g (0.2 wt% CNF)	0.48 g	20 g	0.18 g
PVA-CNF-AlCl3-0.125	3.6 g	20 g (0.2 wt% CNF)	0.6 g	20 g	0.18 g
PVA-CNF	3.6 g	20 g (0.2 wt% CNF)	0.6 g	20 g(H2O)	0.18 g

## Data Availability

The data presented in this study are available in the article. The datasets generated during and/or analyzed during the current study are available from the corresponding author upon reasonable request.

## Sample Availability

Not available.
